# How Good Are Simplified Models for Protein Structure Prediction?

**DOI:** 10.1155/2014/867179

**Published:** 2014-04-29

**Authors:** Swakkhar Shatabda, M. A. Hakim Newton, Mahmood A. Rashid, Duc Nghia Pham, Abdul Sattar

**Affiliations:** ^1^Institute for Integrated and Intelligent Systems (IIIS), Griffith University, 170 Kessels Road, Nathan, QLD 4111, Australia; ^2^Queensland Research Laboratory, National ICT of Australia (NICTA), GPO Box 2434, Brisbane, QLD 4001, Australia

## Abstract

Protein structure prediction (PSP) has been one of the most challenging problems in computational biology for several decades. The challenge is largely due to the complexity of the all-atomic details and the unknown nature of the energy function. Researchers have therefore used simplified energy models that consider interaction potentials only between the amino acid monomers in contact on discrete lattices. The restricted nature of the lattices and the energy models poses a twofold concern regarding the assessment of the models. Can a native or a very close structure be obtained when structures are mapped to lattices? Can the contact based energy models on discrete lattices guide the search towards the native structures? In this paper, we use the protein chain lattice fitting (PCLF) problem to address the first concern; we developed a constraint-based local search algorithm for the PCLF problem for cubic and face-centered cubic lattices and found very close lattice fits for the native structures. For the second concern, we use a number of techniques to sample the conformation space and find correlations between energy functions and root mean square deviation (RMSD) distance of the lattice-based structures with the native structures. Our analysis reveals weakness of several contact based energy models used that are popular in PSP.

## 1. Introduction

Proteins are one of the most important organisms in a living cell, virtually participating in almost every process within the cell including carrying oxygen (by hemoglobin), signaling cells (by insulin), fighting infection (by antibodies), and performing metabolism (by enzymes). For proper functioning, a protein has to fold into a native three-dimensional structure, which is unique, stable, and kinetically accessible [1] in a given environment. However, not much is known about the process of folding. Also, the nature of the energy function is yet unknown. Misfolded proteins cause many critical diseases such as Alzheimer's disease, cystic fibrosis, and mad cow disease. Knowledge about the native structure is of paramount importance, specially for rational drug discovery and to understand the basics of life. Protein structure prediction (PSP) is therefore one of the most challenging problems in biology. Due to slowness and expensiveness of* in vitro* methods, computational methods are of great interest. Given a primary amino acid sequence of protein, the task in PSP is to find its three-dimensional native structure that has the minimum free energy.

In the absence of any known structure or templates,* ab initio* methods guided by a scoring function or energy function are used to predict structures. However, the complexity of searching for a native structure depends on the underlying model. The all-atomic details pose much complexity on the modeling and require huge computational time [4, 5]. Therefore, reduced models are preferred. A general paradigm in PSP [6–8] is to generate simple decoys or candidate structures using a reduced or simplified model and then refine them by adding necessary backbone and side-chain atoms [9, 10]. These reduced or simplified models are also used in investigating the protein folding process in detail [11, 12] and also in the CASP competition by one of the best performing systems such as TASSER [6].

The simplified models are often based on contact based statistical energy models on discrete lattices (cubic or face centered cubic (FCC)). Contact based energy models [13–16] consider interaction energy only among the amino acid types that are in contact. Moreover, the discrete lattices restrict the backbone atoms only to valid lattice points. Such restricted behavior of both the energy models and the lattices poses a twofold concern for the researchers about the goodness of such reduced models. Firstly, how close we are able to model the native structures of the proteins using discrete lattices [17]? Close fits of the backbone of the native structure provide an estimate of the optimal achievable target on discrete lattices and definitely are an indication of the goodness of the lattices being used. Secondly, how well we can guide the search towards the native structures by using the contact based energy models [13–16] regardless of the particular lattice being used? An energy model provides effective search guidance when the structures having lower energy values also have more similarity with the native structures. This proximity or similarity with the native structure is measured by using root mean square deviation (RMSD). Lower RMSD values indicate that structures are more similar to the native one.

In this paper, we address the first concern mentioned above by using the protein chain lattice fitting (PCLF) problem. We developed a constraint-based local search algorithm for the PCLF problem for cubic and face-centered cubic lattices and found very close lattice fits for the native structures. Our algorithm starts with a greedy chain growth algorithm and in subsequent iterations improves by taking moves from the neighborhood generated by a set of operators. On a set of 1192 proteins from the PISCES [18] benchmark set, we achieve average RMSD distances of 1.87 Å and 1.23 Å, respectively, for cubic and FCC cubic lattices. By doing these, we reconfirm the rationale behind selecting these discrete lattices in PSP. For the second concern, we use a number of techniques to sample the conformation space and find correlations between energy functions and root mean square deviation (RMSD) distance of the lattice-based structures with the native structures. The sampling techniques include minimizing RMSD, minimizing the energy functions, and performing a random walk on the conformation space. Using one million samples for each protein and each sampling method, we performed Spearman rank correlation test and found that energy functions such as HP, Barrera, and MJ matrix have mostly negative correlation with the RMSD values. These analyses thus reveal weakness of these contact based energy models, even though they are popular in PSP.

The rest of the paper is organized as follows: a brief summary of the related work in the literature is presented in Section 2; the materials and methods are described in Section 3; experimental results and discussion are presented in Section 4; and the paper is concluded in Section 4 providing a summary of the work and possible future directions.

## 2. Related Work

To the best of our knowledge, no significant study has been performed that has evaluated the effectiveness of the simplified models in terms of the ability of the lattice models to represent native structures and in terms of ability of the energy models to guide the search. Researchers have tried to compare the effectiveness of the lattice models with other models [19] and their effectiveness in folding simulation [20]. However, in the latter case, the studies are conducted using simple two-dimensional lattices only. We address the problem of accuracy of lattices from a point of view of representation to show the accuracy level of the lattices up to which they can model the real proteins. We first explore the literature of the PCLF problem and then that of the energy models.

PCLF problem is proved to be NP-complete [21]. Several techniques have been applied to solve the problem such as exhaustive full enumeration [22], dynamic programming [23], chain growth algorithms [2, 24], move based local search [25], and specialized force fields [26, 27]. Recently Mann et al. [3] proposed LatFit, a tool for PCLF problem for both backbone and side-chain atoms, and achieved the state-of-the-art results. They also proposed another refinement algorithm [28] based on constraint programming techniques.

Contact based energy models [13–16] with discrete lattices have been used extensively in the literature of PSP [11]. A general trend is to optimize the energy function by using various search techniques such as genetic algorithms [29], constraint programming [30, 31], simulated annealing [32, 33], and memory based methods [34, 35]. However, it is important to assess their performance on a wide variety of protein sequences to see if they really work for simplified models. In the literature, quasichemical approximation techniques to derive contact based energy functions like Miyajawa-Jernigan matrices [14] are criticized for neglecting the peptide bonding of the amino acids [36]. However, no comprehensive study was found in the literature to show the effectiveness of such energy functions. The ability of contact based energy methods to discriminate the native state from the decoys was investigated in [37, 38] and a general negative answer was found. Later on, a new empirical energy model was presented in [16] that was much simpler than the Miyajawa-Jernigan matrices and was successfully able to discriminate the native state from the decoy sets. These energy functions are used by the researchers both in fold recognition [39] and in* ab initio* methods to guide the search [31].

In this paper, we use a constraint-based local search to produce state-of-the-art results for PCLF problem and thus show the effectiveness of cubic and FCC cubic lattices. Using a number of guided sampling techniques, we also perform an assessment of the effectiveness of different energy models. The analysis reveals weakness of those energy functions.

## 3. Materials and Methods

Proteins are polymers of amino acid monomers. There are 20 different amino acids. In a simplified model, all monomers have an equal size and all bonds are of equal length. Each monomer is modeled by a point in a three-dimensional lattice (*lattice* constraint). The given amino acid sequence fits into the lattice: pair of all consecutive amino acids in the sequence are also neighbors in the lattice (*chain* constraint) and two monomers cannot occupy the same point in the lattice (*self-avoiding* constraint). A simplified energy function is used in calculating the energy of a structure.

**Figure 1:**
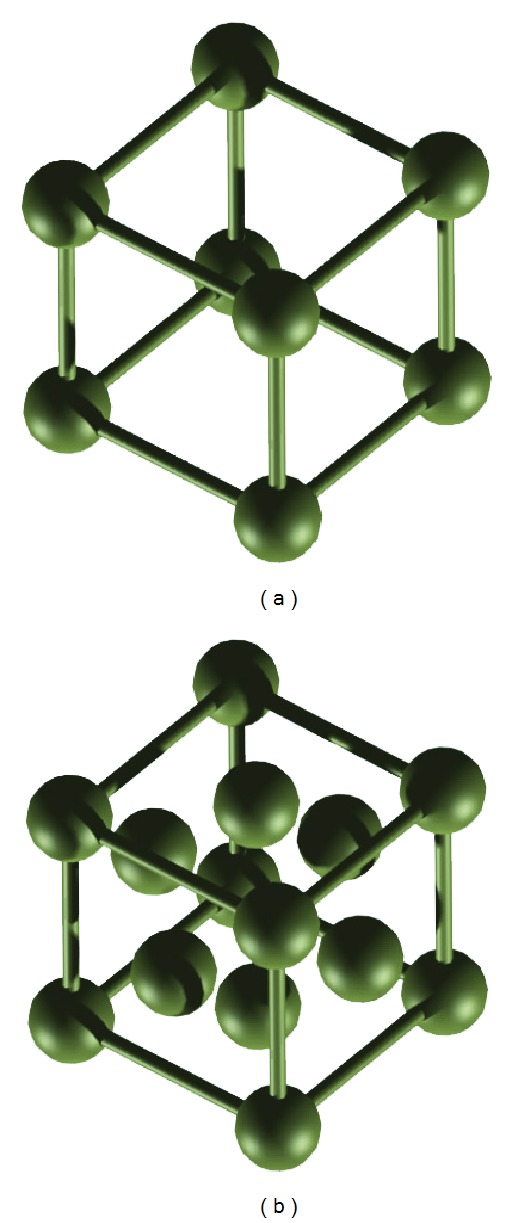
Different 3D lattices: (a) cubic and (b) FCC lattice.

Two lattice points *p*, *q* *ϵ* *L* are said to be in* contact* or* neighbors* of each other, if $q=p+{\vec{v}}_{i}$ for some vector ${\vec{v}}_{i}$ in the basis of *L*. There exist a number of lattice models (see Figure 1). FCC lattice is preferred to cubic lattice since the former provides a higher degree of freedom for placing an amino acid and has a higher packing density [40]. The points on FCC lattice are generated by 12 basis vectors: ${\vec{v}}_{1}=(1,1,0)$, ${\vec{v}}_{2}=(-1,-1,0)$, ${\vec{v}}_{3}=(-1,1,0)$, ${\vec{v}}_{4}=(1,-1,0)$, ${\vec{v}}_{5}=(0,1,1)$, ${\vec{v}}_{6}=(0,1,-1)$, ${\vec{v}}_{7}=(0,-1,1)$, ${\vec{v}}_{8}=(0,-1,-1)$, ${\vec{v}}_{9}=(1,0,1)$, ${\vec{v}}_{10}=(-1,0,1)$, ${\vec{v}}_{11}=(1,0,-1)$, and ${\vec{v}}_{12}=(-1,0,-1)$, and the points on a cubic lattice are generated by 6 basis vectors: ${\vec{v}}_{1}=(1,0,0)$, ${\vec{v}}_{2}=(-1,0,0)$, ${\vec{v}}_{3}=(0,1,0)$, ${\vec{v}}_{4}=(0,-1,0)$, ${\vec{v}}_{5}=(0,0,1)$, and ${\vec{v}}_{6}=(0,0,-1)$.

### 3.1. Constraint Programming Model for Protein Structures

In our constraint programming model, we are given a sequence *S*, where each element *s*_*i*_ ∈ *S* is an amino acid type. Each amino acid *i* is associated with a point *p*_*i*_ = (*x*_*i*_, *y*_*i*_, *z*_*i*_) ∈ *Z* ^3^. The decision variables are the *x*, *y*, and *z* coordinates of a point. For a sequence of length *n*, the domain of the variables is the range [−*n*, *n*]. Formally, ∀_*i*_ *x*_*i*_ ∈ [−*n*, *n*], ∀_*i*_ *y*_i_ ∈ [−*n*, *n*], and ∀_*i*_ *z*_*i*_ ∈ [−*n*, *n*]. The first point is assigned as (0,0, 0), which is a valid point in the FCC lattice. The rest of the points follow the constraint, $\forall_{i<n}({\vec{a}}_{i})\in \{{\vec{v}}_{1},\ldots ,{\vec{v}}_{12}\}$. Here, ${\vec{a}}_{i}$ is the absolute vector between points (*x*_*i*+1_, *y*_*i*+1_, *z*_*i*+1_) and (*x*_*i*_, *y*_*i*_, *z*_*i*_), and $\left\{{\vec{v}}_{1},\ldots ,{\vec{v}}_{12}\right\}$ are the basis vectors for FCC lattice. Thus all points satisfy the* lattice* constraint and* chain* constraint. The* self-avoiding* constraint is defined using the all-different constraint, all-different(∀_*i*_ *p*_*i*_). The all-different constraint is defined over a set of points and it is satisfied only if no two elements are the same in the set. We define sqrdist(*i*, *j*) as the square of Euclidean distances between two points *p*_*i*_ and *p*_*j*_. Now, contact(*i*, *j*) = 1, if sqrdist(*i*, *j*) = 2, and contact(*i*, *j*) = 0, if sqrdist(*i*, *j*) ≠ 2.

### 3.2. Intramolecular Similarity Measure

The choice of distant measure is very important for our experiments. Distance measures between two structures cRMSD are calculated by taking the square root of the average distance between corresponding atoms of two structures. However, in case of the the molecular structures sampled from molecular dynamics or other forms of sampling, often the structure drifts away from the origin and rotates in an arbitrary way. Calculating cRMSD requires finding an optimal alignment of two structures first and then calculating RMSD. Moreover, in cases where we wish to find structures that are similar to each other in potential energy (free energy), cRMSD will find a structure with overall minimum average atomic displacement by treating all atoms equally. However, in cases like protein structure prediction, we cannot treat all atoms similarly since atoms on the outside of the protein can often move without affecting the potential energy, while atoms at the centers have more impact on the energy function even for slightest movements. For these reasons, intramolecular distance measures like dRMSD are developed to address the shortcoming of cRMSD based measures. For two given structures *C* = *p*_1_,…, *p*_*n*_ and *B* = *b*_1_,…, *b*_*n*_, dRMSD is defined as follows:

(1) dRMSD(B,C)=∑i<j(dist(bi,bj)−dist(pi,pj))2n∗(n−1)/2,

where function dist⁡(*p*_*i*_, *p*_*j*_) denotes the Euclidean distance between two points *p*_*i*_ and *p*_*j*_.

### 3.3. Problem Definition

Now, given the native structure of a protein in full atomic representation and the backbone of the given native structure *B* = *b*_1_,…, *b*_*n*_, in PCLF problem, the task is to find a structure in the lattice, *C* = *p*_1_,…, *p*_*n*_ such that the distance between *B* and *C* is minimized. The backbone of a protein structure is defined by the *α*-Carbon positions. In order to normalize, we consider neighborhood distance in the discrete lattices (1 in case of cubic and $\sqrt{2}$ in case of FCC) to be equal to 3.8 Å, which is the average distance between two consecutive *α*-Carbon atoms in real proteins. This distance is enough to avoid possible steric clashes after adding other atoms during refinement. In PCLF problem, we wish to minimize dRMSD defined in (1). The objective function becomes

(2) $$\begin{array}{c} \text{obj}=\text{Mimimize}\;\text{dRMSD}\left(B,C\right). \end{array}$$

### 3.4. Search Procedure

The optimization for PCLF starts with a chain growth initialization technique. The chain growth initialization is greedy in nature. It starts by assigning (0,0, 0) to the first amino acid position. For each of the next positions, it calculates the new point using the possible basis vectors for the selected lattice type, and if that position is not occupied, it also calculates the partial dRMSD value for the assigned positions (*p*_0_,…, *p*_*i*_) only. It greedily selects the basis vector that results in the minimum dRMSD. Ties are broken by a predefined order using a FIFO data structure. Pseudocode for selection of direction is given in Algorithm 2. If no free positions are available, the algorithm backtracks and starts from the last position. It also keeps track of the directions once set to a position and skips those when backtracking. The pseudocode of the algorithm is given in Algorithm 1. This chain growth initialization produces initial structures with very low dRMSD values (see Table 1).

The pseudocode for the search is given in Algorithm 3. At each iteration, we randomly select an operator. According to the selected operator type, points are selected randomly. We maintain a tabu list of recently used moves. We use two operators: jump move operator (see [41] for details) and pull move operator proposed in [42]. After the selection of the points, the substructures comprising those positions are reoptimized by allowing all possible valid orientations by jump move or pulled in all possible directions in the neighborhood by using pull moves. In case of jump moves, multiple points are selected depending on the parameter *move* *Size*. Initially, *move* *Size* is set to 1. For a pull move, a single point that is not in the tabu list is selected. After selecting the points, the neighborhood moves are generated using the selected operator. After generation, all the candidate moves are simulated. Simulation of a move temporarily calculates the changes in the heuristic functions without committing the move. After simulation, only the best candidate is selected. The selection is based on the dRMSD value only. However, we always include the current structure as a candidate and the search progresses monotonously in a nonincreasing manner. If the search is not able to find improvement in the global minimum for a number of steps determined by the parameter *stagnation*, we increase the *move* *Size* by one and the parameter *stagnation* is also multiplied by a *factor*. They are set to initial values whenever there is an improvement.

### 3.5. Energy Models

The contact based energy functions are generally used along with lattices in the simplified models. We analyzed three different energy functions proposed in [13, 14, 16]. Formally, energy of a structure *C* in the setting of simplified models described in Section 3 is defined by the following equation:

(3) $$\begin{array}{c} E\left(C\right)=\overset{}{\underset{i<j}{\sum }}contact\left(i,j\right)\times energy\left(s_{i},s_{j}\right). \end{array}$$

Here, energy(*s*_*i*_, *s*_*j*_) is defined by the particular energy interaction of the amino acid types, *s*_*i*_, *s*_*j*_ in a given sequence *S*, for a particular energy model. The first energy model, denoted by hp-basic in this paper, is the basic HP energy model proposed in [13]. It considers only the interaction between hydrophobic residues. In other words, there is an energy potential, −1, defined only for hydrophobic interactions. The other two energy models are elaborate in nature and 20 × 20 energy models considering all types of interaction between amino acids. They are denoted as mj [14] and bre [16] throughout this paper. Details of these energy matrices are given in [14, 16].

### 3.6. Sampling Algorithm

Sampling the conformation space is important for the second part of our experiment. Since we attempt to find the native structure, we first study the energy values of the structures that are visited when we minimize the dRMSD value. By minimizing dRMSD using our PCLF algorithm in Algorithm 3, we are able to generate sample structures that are within very close proximity of the native structures (experimental results show this). However, in reality the dRMSD values will be unknown and we have to minimize the energy values with the hope that lower energy values will lead to lower dRMSD structures. We therefore also generate sample protein structures by running a search algorithm that minimizes a given energy function. For this, we use Algorithm 3 but with dRMSD being replaced by the given energy function. Lastly, we also use a random walk algorithm that starts with a random initialization and in each iteration picks a random candidate from the randomly generated neighbor structures. We do not use a sampling method that randomly generates all structures. For each lattice type, we therefore have 5 different search algorithms to generate samples.

1. PCLF search: this algorithm is essentially the algorithm presented in Algorithm 3. The search is directly guided by dRSMD. At each iteration, dRSMD is minimized and we reported dRSMD values and values of the energy functions for all selected candidates.
2. Guided search, hp-basic: this algorithm is similar to that presented in Algorithm 3 except in Line 1 and Line 10. The search is guided by the basic HP energy model [13]. In each iteration, we reported values of all the energy functions and dRSMD values of the selected candidate structures.
3. Guided search, mj: this algorithm is similar to that presented in Algorithm 3 except in Line 1 and Line 10. The search is guided by MJ Matrix model proposed in [14]. In each iteration, we reported values of the energy function and dRSMD values of the selected candidate structures.
4. Guided search, bre: this algorithm is similar to that presented in Algorithm 3 except in Line 1 and Line 10. The search is guided by the empirical energy model proposed in [16]. In each iteration, we reported values of the energy function and dRSMD values of the selected candidate structures.
5. Random walk: we ran another version using no guidance and selecting the candidates randomly from the list of the generated structures. This algorithm is essentially a random walk in the space of feasible structures. In each iteration, we reported values of all the energy functions and dRSMD values of the selected candidate structures.

### 3.7. Implementation

We implemented our framework using C++ on top of the constraint-based local search system, Kangaroo [43]. The constraints are maintained by invariants in kangaroo. Invariants are special constructs that are defined by using mathematical operators over the variables. Simulation of moves, execution, and necessary propagation of constraint and function values are performed incrementally using Kangaroo.

## 4. Results and Discussion

We ran our experiments on the Gowonda clusters provided by Griffith University with nodes equipped with Intel Xeon CPU X5650 processors @2.67 GHz, QDR 4 x InfiniBand Interconnect. Experiments were run on benchmark protein sequences taken from the PISCES [18]. These proteins are originally used in [3]. The proteins were selected by enforcing 40% sequence identity cutoff, with chain length 50–300, *R*-factor ≤0.3, and resolution ≤ 1.5 Å to derive a high-quality set of proteins. The resulting benchmark set contains 1192 proteins exhibiting a mean length of 160 (*σ* = 64).

### 4.1. Accuracy of Lattice Fitting

We ran our algorithm on both cubic and FCC lattices. For each protein sequence in the benchmark set, we ran our algorithm 5 times with a timeout of 1 hour. Average dRMSD values are reported in Table 1. We report average dRMSD values produced by the greedy chain growth algorithm in the “initial” column and the final average values in the “final” column. Since we consider backbone-only structures denoted by *α*-Carbon atoms, we compare our results with those in the literature with backbone-only models. Average dRMSD values produced by the algorithm by Park and Levitt [2] and LatFit program [3] are reported as in [3]. The values reported in Table 1 show that our approach produces lattice fits with lower dRMSD values. Percentage improvements of our approach over other methods are also shown in Table 1 in column “Imp.” Improvements are defined as Imp. = 100 × (Average dRMSD of our approach-Average dRMSD of other approach)/Average dRMSD of other approach. The differences in the dRMSD value are statistically significant since the number of samples is large and we take average values of 5 runs. However, we performed statistical *t*-test with 95% significance level to ensure the significance of these results. From the lower dRMSD values reported for both types of lattices, we conclude that with these types of discrete lattice, it is possible to generate structures that are within close proximity of the native structures. The state-of-the-art methods for structure prediction confirm this value for the backbone models and also for any other detailed models [8, 9]. In other words, these discrete lattices provide realistic backbone for real protein structures.

### 4.2. Effectiveness of Energy Models in Search Guidance

*Ab initio* search based PSP algorithms usually minimize a given energy function. The prior assumption is that if the energy model is effective, then the structures with lower energy values have lower dRSMD values too. In other words, minimizing the energy function would result in minimizing dRSMD value and the search will eventually lead to structures closer to the native structure. In that case, the values of the energy functions must show a strong positive correlation with dRSMD. We ran each of 5 variants of sampling algorithms mentioned in Section 3.6 and generated one million sample structures during the search for each type of lattice. After finishing each run, we test correlation between the dRSMD values and values of each of the energy functions reported for the candidate structures of each protein. We performed Spearman rank correlation test for each protein.

**Figure 2:**
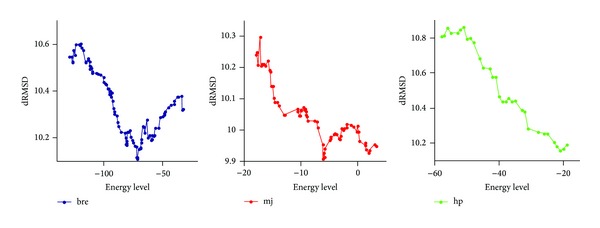
Plot of values of three different energy function values of the structures generated for 1A6M against their distant root mean square deviation (dRMSD) values found by the guided sampling algorithms.

The summary of the results is given in Table 2. From the bold-faced values in Table 2, we see that the search when guided by the energy functions shows strong negative correlations (<−0.5) for most proteins, while a positive correlation was ideally desired. Typical plots of dRSMD against the energy function values are shown in Figure 2 for the protein* 1A6M*. For this protein, we see that, for each of the energy functions, dRSMD value does not decrease with the minimization of the energy values. Similar plots are also found for other protein sequences as well. Thus we conclude that the search when guided by the energy functions, that is, minimizing the energy value does not result in minimizing the dRSMD values.

In Table 2, we also notice that the correlation coefficients are negative or weakly positive (<0.5) for most of the proteins even when the sampling was performed by minimizing dRSMD. These observations are indicative only because in effect we will not minimize dRMSD. Weak positive correlations are also found for two of the energy functions for the samples generated by random walk. In order to test the significance of the weak positive correlations, we further analyze the quality of the structures that were generated by our sampling algorithms.

**Figure 3:**
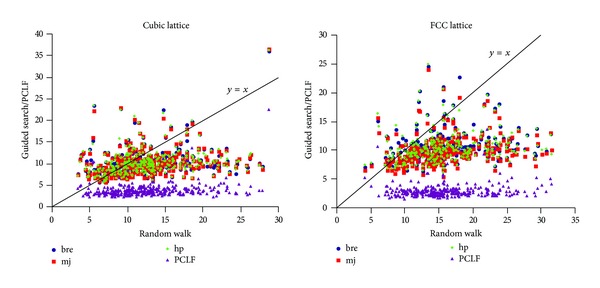
Scatter plot of minimum dRSMD values found by each of the sampling algorithms against the minimum dRSMD value found by random walk.

Figure 3 shows the scatter plots of minimum dRSMD values found for each protein by each of the sampling algorithms against the minimum dRSMD value found by random walk on both types of lattices. It is clearly visible that the PCLF search produces structures with lower dRSMD (in the plot near the *x*-axis) compared to other methods and thus covers the areas of the search spaces that are closer to the native structures. For the sampling methods that minimize energy functions, most proteins in the chart lie below *x* = *y* line meaning that the minimum dRSMD values found by them are better than the minimum dRSMD values found by the random walk. This further means that the random walk based sampling method does not generate structures that are closer to the native structures and so the weak positive correlations are not very significant. Thus a random walk instead of using energy functions as search guidance would not be useful. Overall these observations reveal the weakness of the energy functions used in this experiment; that is, minimizing them does not necessarily mean guide the search on the way to the native structures.

## 5. Conclusions

In this paper, we propose a constraint-based local search framework that produces state-of-the-art results for the protein chain lattice fitting (PCLF) problem for real proteins. This confirms the effectiveness of using discrete lattices in protein structure prediction (PSP). In addition to this, we also analyze several simplified energy functions and their effectiveness to find better structures in terms of dRMSD values. Our analysis revealed the weakness of several contact based energy models used in the literature of PSP. The algorithm that we use to find the lattice fits and sampling for the structures is stochastic in nature. In future, we wish to add side chains and secondary motifs and use the discrete lattices effectively for PSP guided by effective energy functions and the heuristics. We also wish to develop a web server providing the service of lattice fitting and extend our work to other lattices as well.

## Figures and Tables

**Algorithm 1:**
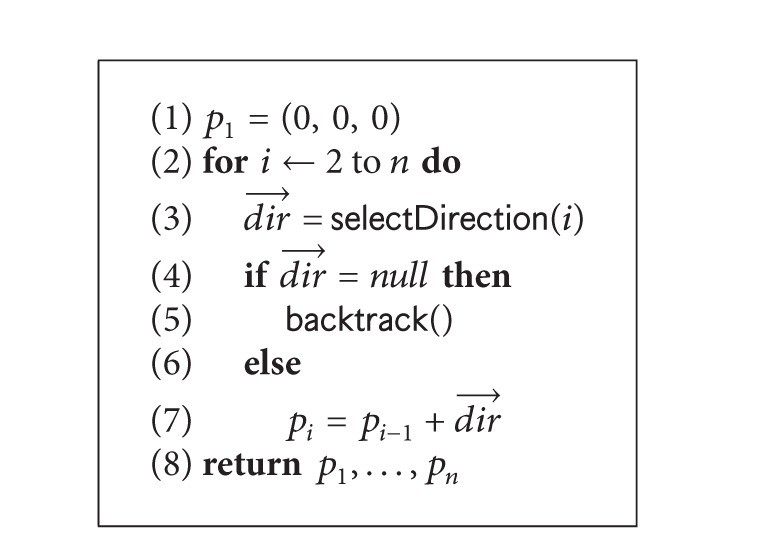
chainGrowthInitialize().

**Algorithm 2:**
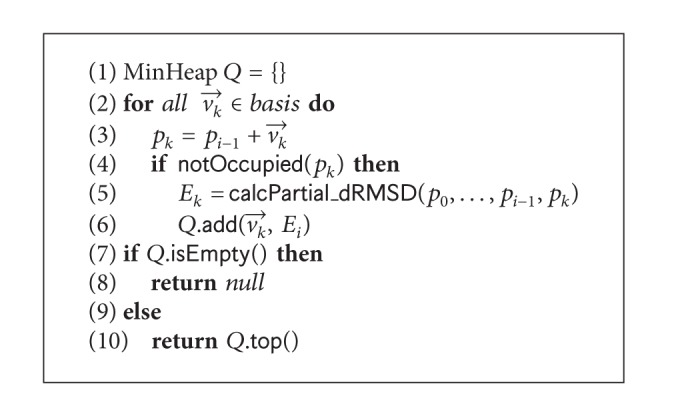
selectDirection(*position* *i*).

**Algorithm 3:**
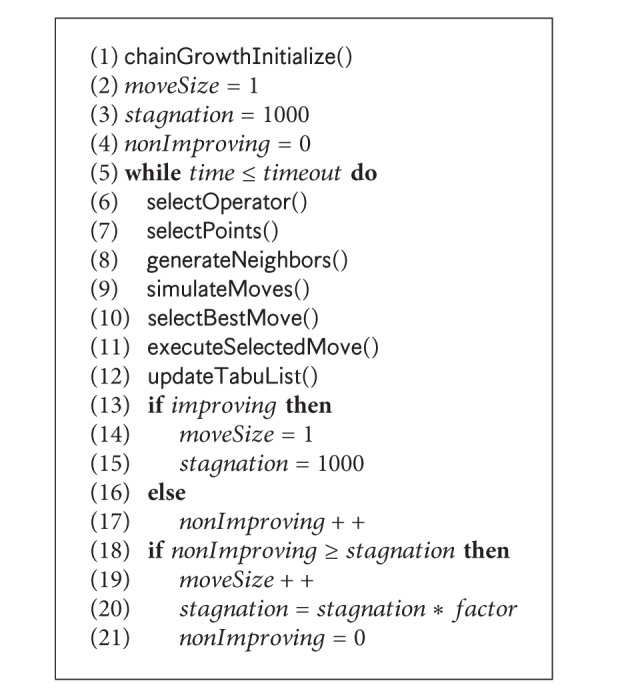
PCLFSearch().

**Table 1:** Average distance root mean square deviation (dRSMD) values achieved for the benchmark protein sequences in five runs for different algorithms in the literature and percentage improvements produced by our approach over other approaches.

Lattice type	Park and Levitt [2]		Mann et al. [3]		Our approach	
	Avg.	Imp.	Avg.	Imp.	Initial	Final
Cubic	2.34	20.08%	2.08	10.09%	2.86	** 1.87**
FCC	1.46	15.75%	1.34	8.21%	2.03	** 1.23**

**Table 2:** Proportion of the number of protein sequences that fall in the different ranges of correlation coefficient between energy function value and distant root mean square deviation (dRSMD), produced by different sampling techniques: sampled by PCLF search, sampled by energy function guidance, and sampled by random walk.

Energy function	Correlation coefficient for cubic lattice (%)				Correlation coefficient for FCC lattice (%)			
	>0	>0.5	≤0	<−0.5	>0	>0.5	≤0	<−0.5
Sampling by using PCLF search								
hp-basic	11.56	2.02	88.43	58.67	5.73	0.28	94.26	74.21
mj	70.20	24.35	29.79	6.59	73.72	28.85	26.28	6
bre	9.16	0.57	90.83	66.47	3.71	0	96.28	80

Sampling by using guided search								
hp-basic	9.88	5.14	** 90.12**	** 79.05**	3.69	0.86	** 96.31**	** 90**
mj	31.85	16.38	** 68.15**	** 51.63**	19.31	5.42	** 80.69**	** 51.84**
bre	2.09	1.43	** 97.91**	** 93.98**	0.86	0.22	** 99.14**	** 93.49**

Sampling by using random walk								
hp-basic	86.22	1.04	13.78	0.13	87.54	1.23	2.36	0.11
mj	31.76	0.13	68.24	0	54.25	1.18	46.75	0.24
bre	85.30	2.62	14.70	0.26	81.28	1.28	18.72	1.54
